# cAMP/PKA signaling in endocrine hypertension: genetic mechanisms and pathophysiological insights

**DOI:** 10.3389/fendo.2026.1748702

**Published:** 2026-02-24

**Authors:** Jose Antonio B. Lima Sobrinho, Madson Q. Almeida

**Affiliations:** 1 Unidade de Adrenal, Laboratório de Endocrinologia Molecular e Celular Laboratório de Investigação Médica 25' (LIM25), Divisão de Endocrinologia e Metabologia, Hospital das Clínicas, Faculdade de Medicina da Universidade de São Paulo, São Paulo, Brazil; 2 Unidade de Oncologia Endócrina, Instituto do Câncer do Estado de São Paulo (ICESP), Faculdade de Medicina da Universidade de São Paulo, São Paulo, Brazil

**Keywords:** cAMP, Cushing syndrome, endocrine hypertension, phosphodiesterase, primary aldosteronism, protein kinase A

## Abstract

The cyclic adenosine monophosphate (cAMP)–protein kinase A (PKA) signaling pathway plays a central role in adrenal function, steroidogenesis, and blood pressure regulation. Increasing evidence suggests that dysregulation of this pathway contributes to several forms of hypertension, both endocrine and non-endocrine. A growing number of germline and somatic alterations affecting components of the cAMP/PKA axis have been implicated as key drivers of hypertensive disorders. Among these, activating pathogenic variants (PV) in *GNAS*, which encodes the stimulatory G protein α-subunit (Gsα) responsible for cAMP production, have been linked to cortisol excess. Mosaic *GNAS* PV *cause McCune-Albright syndrome*, which may present with ACTH-independent Cushing syndrome, while somatic *GNAS* PV have been identified in cortisol-producing adrenal adenomas. Germline inactivating variants in *PRKAR1A* are associated with *Carney complex and primary pigmented nodular adrenocortical disease (PPNAD).* Furthermore, germline alterations in phosphodiesterases such as *PDE11A* and *PDE8B*, which impair cAMP degradation, have been associated with Cushing syndrome and micronodular adrenal hyperplasia. Somatic activating PV in *PRKACA*, the gene encoding the catalytic subunit of PKA, have also been described in cortisol-producing adenomas. In primary aldosteronism, recent studies—including data from our group—suggest that germline variants in *PDE2A* and *PDE3B* may contribute to bilateral adrenal hyperplasia and autonomous aldosterone production by modulating intracellular cAMP levels. Additionally, gain-of-function PV in *PDE3A* have been associated with a familial form of salt-independent hypertension characterized by enhanced PKA signaling and vascular remodeling. This expanding body of evidence underscores the critical role of the cAMP/PKA pathway in the pathophysiology of distinct hypertensive phenotypes and highlights novel molecular mechanisms and potential therapeutic targets that merit further investigation.

## 1 Introduction

### 1.1 Hypertension and the role of second messengers

Systemic arterial hypertension constitutes one of the main risk factors for cardiovascular and renal diseases, reflecting heterogeneous pathophysiological mechanisms (1–3). Although the primary form, of multifactorial origin, predominates in most cases, secondary hypertension occurs in a subset of patients (around 10% of cases) in whom specific causes can be identified (4, 5). Among these, endocrine hypertension stands out for resulting from autonomous hormonal secretion, which directly interferes with molecular pathways regulating blood pressure (6). Alterations in receptors, intracellular signaling cascades, and transcription factors represent critical points in this process, in which the aberrant activation of secondary messengers establishes the connection between genetic alterations, whether germline or somatic, and the development of the hypertensive phenotype (4–7).

Second messenger pathways play fundamental roles in cellular physiology, converting and amplifying extracellular signals, such as hormones and neurotransmitters, into intracellular responses that, depending on the specific tissue, can regulate factors such as growth, differentiation, metabolism, and cell survival (8, 9). Among these messengers is adenosine 3’,5’-cyclic monophosphate (cAMP), which plays a central role across multiple tissues and physiological contexts, exerting effects that range from regulation of gene transcription to the control of metabolic and cardiovascular functions (10–12).

### 1.2 The cAMP-PKA pathway and its regulatory mechanisms

**Figure 1:**
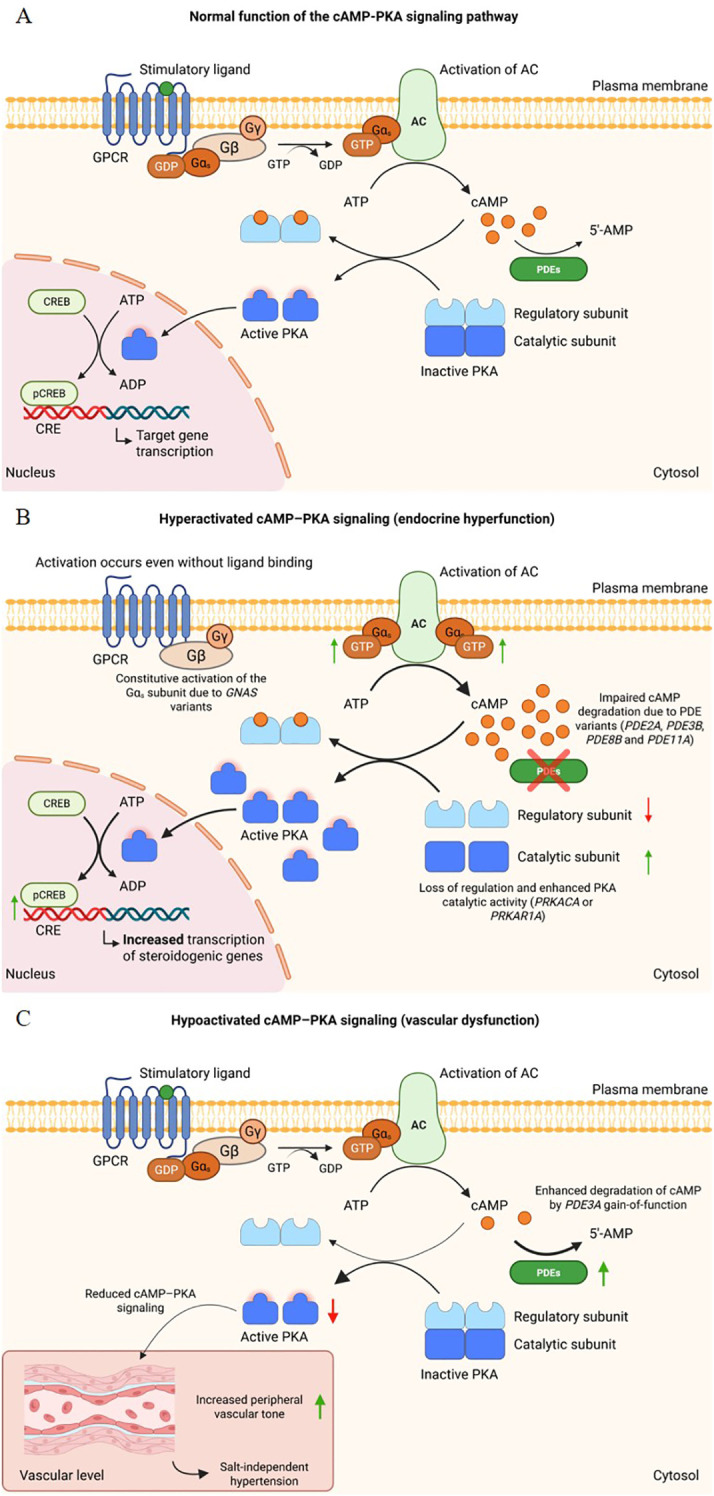
Normal function and dysregulation of the cAMP–PKA signaling pathway. **(A)** Schematic representation of the physiological activation of the cAMP–PKA pathway. Ligand binding to the stimulatory G protein–coupled receptor (GPCR) activates the Gα_𝑠_ subunit, promoting GDP–GTP exchange and stimulating adenylate cyclase (AC), which increases intracellular cAMP levels. cAMP triggers the dissociation of protein kinase A (PKA), releasing its catalytic subunits that phosphorylate nuclear targets such as CREB, thereby regulating gene transcription. Phosphodiesterases (PDEs) hydrolyze cAMP and maintain signaling homeostasis. **(B)** Dysregulation of the pathway caused by genetic alterations in key regulatory components. Constitutive activation of Gα_𝑠_ (*GNAS*) enhances cAMP production independently of receptor stimulation. Impaired function of the PKA regulatory subunit (*PRKAR1A*) decreases inhibition of catalytic activity, whereas activating changes in the catalytic subunit (*PRKACA*) increase basal kinase activity. Dysfunction of PDEs reduces cAMP degradation. These abnormalities converge to hyperactivate PKA, augment CREB phosphorylation, and amplify transcription of downstream target genes, contributing to autonomous steroidogenesis and adrenocortical hyperplasia. **(C)** Pathway hypoactivation associated with enhanced cAMP degradation. Gain-of-function variants in *PDE3A* increase cAMP hydrolysis, reducing intracellular cAMP availability and downstream PKA activity. In vascular smooth muscle cells, reduced cAMP–PKA signaling contributes to increased peripheral vascular tone and salt-independent hypertension, as observed in hypertension with brachydactyly.

The cAMP-mediated signaling pathway begins when an extracellular stimulus binds to G protein-coupled receptors (GPCRs), promoting activation of adenylyl cyclase (AC) and culminating, through a cyclization reaction, in the conversion of ATP into cAMP (**Figure 1A**) (8, 9, 13). Among the various effectors modulated by this nucleotide, protein kinase A (PKA) is responsible for triggering multiple cellular responses (9, 13). The PKA holoenzyme is a heterotetramer consisting of two regulatory and two catalytic subunits, which assemble into type I or type II isoforms depending on the regulatory subunit involved (14). Once activated by cAMP binding to the regulatory subunits, which promotes the release of the catalytic subunits, PKA phosphorylates different target proteins, regulating processes such as hormone secretion, steroidogenesis, vascular tone, and gene expression through transcription factors such as cAMP response element-binding protein (CREB) (10, 15, 16). In this way, the cAMP-PKA pathway constitutes a point of convergence for multiple hormonal and mechanical stimuli, playing an essential role in endocrine and cardiovascular homeostasis (8, 17).

Proper functioning of the cAMP-PKA pathway also depends on the action of phosphodiesterases (PDEs), enzymes that hydrolyze cyclic nucleotides and thereby ensure both compartmentalization and temporal control of signaling by modulating the availability of cAMP for PKA (13, 18). Within this pathway, PDEs act not only as signal terminators but also as central regulators, directing the distribution of cyclic nucleotides among different effectors such as cyclic nucleotide-gated channels, cAMP-activated exchange proteins (cAMP-GEFs), and the extent of PKA activation itself (19).

Beyond their function in regulating the cAMP-PKA cascade, PDEs are also modulated by other second messengers and kinases, functioning as integrators that connect the cyclic nucleotide system to additional signaling pathways. Through their localized activity, they contribute to intracellular compartmentalization by restricting cAMP diffusion within distinct cellular microdomains (19). In humans, PDEs are encoded by 21 genes and, due to alternative mRNA splicing events, give rise to more than 100 isoforms grouped into a superfamily of 11 distinct PDE families (20). This structural and functional diversity, resulting from complex genetic organization, enables precise modulation of cAMP effects in different cellular and tissue contexts (10).

### 1.3 Genetic and molecular alterations in the cAMP–PKA pathway and endocrine hypertension

Impairment of this regulatory mechanism controlling cAMP degradation leads to signaling alterations that have been implicated in multiple endocrine and cardiovascular diseases, such as hypertension (21–23). These pathogenic mechanisms may result either from gain-of-function pathogenic variants (PV), such as those in *PDE3A* that increase cAMP hydrolysis and underlie hypertension with brachydactyly type E (20, 24), or from loss-of-function variants, as described for *PDE11A* and *PDE8B*, which impair enzymatic activity and lead to aberrant cAMP accumulation in adrenal cells, predisposing to adrenocorticotropic hormone (ACTH)-independent macronodular or micronodular adrenal hyperplasia and Cushing syndrome (CS) (21, 25, 26). In addition, germline variants affecting *PDE2A* and *PDE3B* have been reported in patients with bilateral primary aldosteronism (PA), further expanding the spectrum of PDE defects associated with endocrine hypertension (27). Collectively, these disorders illustrate how genetic defects in distinct PDEs, by either enhancing or reducing cAMP hydrolysis, disrupt intracellular homeostasis, drive aberrant activation of the cAMP-PKA pathway, and ultimately contribute to the pathogenesis of endocrine forms of hypertension.

However, not only alterations in PDEs can lead to hypertension and other endocrine disorders arising from dysfunctions in the cAMP-PKA pathway. Genes that encode central elements of this cascade may also present germline or somatic variants with a direct impact on PKA activity, culminating in autonomous cortisol secretion and the development of ACTH-independent CS, a condition frequently associated with hypertension (7, 28). The *GNAS* gene, which encodes the α subunit of the stimulatory G protein (Gsα), may harbor activating PV that cause persistent increases in cAMP production. These PV occur in a mosaic pattern in McCune–Albright syndrome (MAS) (29, 30) and as somatic events in adrenal cortisol-producing adenomas (31–33). Inactivating alterations in *PRKAR1A*, which encodes the type 1α regulatory subunit of PKA, result in loss of inhibitory control over the enzyme and are associated with Carney complex (CNC) and isolated primary pigmented nodular adrenocortical disease (PPNAD) (34–36). Somatic *PRKACA* activating PV, which lead to constitutive activation of the PKA catalytic subunit, have been identified in cortisol-producing adrenal adenomas (37, 38). A summary of the main genes, their molecular defects, and associated clinical phenotypes is presented in **Table 1**.

**Table 1:** Endocrine hypertension disorders linked to the cAMP–PKA pathway: genes involved and molecular mechanisms.

Gene	Predominant alteration type	Mechanism in the cAMP–PKA pathway	Brief clinical consequence
ACTH-independent Cushing’s syndrome			
*GNAS*	Somatic gain-of-function or germline mosaicism	Constitutive activation of Gsα leading to persistently increased cAMP levels	Adrenocortical adenoma and autonomous cortisol secretion
*PRKAR1A*	Germline loss-of-function	Loss of the regulatory subunit resulting in increased constitutive PKA activity	Isolated or Carney complex–associated PPNAD with autonomous cortisol secretion
*PRKACA*	Somatic gain-of-function (p.L206R) or rare germline amplification	Autonomous activation of the catalytic PKA subunit, independent of *PRKAR1A* regulation	Adenoma and autonomous cortisol secretion
*PDE8B*	Germline loss-of-function/missense	Impaired cAMP degradation leading to PKA hyperactivation	Micronodular adrenal hyperplasia with autonomous cortisol secretion
*PDE11A*	Germline loss-of-function	Impaired cAMP degradation leading to PKA hyperactivation	Micronodular adrenal hyperplasia (including PPNAD) with autonomous cortisol secretion
Primary aldosteronism			
*PDE2A*	Rare germline variants	Impaired cAMP degradation leading to PKA hyperactivation	Possible autonomous aldosterone secretion, renin suppression, and hypertension
*PDE3B*	Rare germline variants	Impaired cAMP degradation leading to PKA hyperactivation	Possible autonomous aldosterone secretion, renin suppression, and hypertension
Hypertension with brachydactyly (Bilginturan syndrome)			
*PDE3A*	Germline gain-of-function	Increased cAMP hydrolysis in vascular smooth muscle reduces vasodilatory PKA signaling and promotes arterial remodeling	Severe, salt-independent hypertension associated with type E brachydactyly

Beyond genetic defects affecting PDEs and core intracellular components of the cAMP–PKA cascade, dysregulation of upstream GPCR signaling may also contribute to aberrant pathway activation in endocrine tissues. In particular, aberrant expression, overexpression, or ectopic activation of GPCRs such as *LHCGR*, *GIPR*, *AVPR1A*, *5-HT4R*, *TRHR*, *GnRHR*, and *GPR101* has been described in a variety of endocrine tumors and hyperplasias, giving rise to illicit ligand-dependent regulatory loops that promote autonomous hormone secretion and cellular proliferation (39). In these contexts, excessive stimulation of the cAMP–PKA pathway reflects abnormal receptor expression patterns and microenvironmental signaling, rather than intrinsic defects of the cAMP-PKA intracellular signaling machinery.

Against this complex regulatory background, the present review focuses on ligand-independent dysregulation of the cAMP–PKA signaling cascade as a primary pathogenic mechanism underlying endocrine hypertension. Specifically, alterations ranging from impaired cyclic nucleotide degradation by PDEs to direct dysregulation of PKA activity mediated by genes such as *GNAS*, *PRKAR1A*, and *PRKACA* ultimately culminate in aberrant pathway activation, autonomous steroidogenesis, and the development of endocrine disorders associated with hypertension. An integrated schematic representation of the physiological and dysregulated states of this pathway, including alterations in these genes, is shown in **Figure 1**.

## 2 Endocrine hypertension and the cAMP-PKA pathway

### 2.1 ACTH-independent Cushing syndrome

#### 2.1.1 Clinical overview

CS corresponds to the set of clinical manifestations resulting from chronic exposure to excessive levels of glucocorticoids (40). Although rare, with an incidence of 0.2–5 cases per million people per year, epidemiological data on CS remain limited, as most prevalence studies primarily focus on Cushing disease (41). Nevertheless, available population-based studies indicate a clear female predominance, with a mean age at diagnosis of approximately 42 years, and a higher prevalence among individuals with resistant hypertension, poorly controlled type 2 diabetes, or early-onset osteoporosis (42–44). In contrast, pediatric cases show an age-dependent sex distribution, with male predominance before puberty that progressively equalizes toward adolescence (45). Clinical manifestations are heterogeneous and often nonspecific, delaying diagnosis for years (46, 47). Among the most suggestive signs are easy bruising, proximal myopathy, violaceous striae, and facial plethora (40). In addition to these changes, excess cortisol markedly affects the cardiovascular system, with arterial hypertension representing one of its main consequences (48). This may present early in young adults or as resistant hypertension, thereby increasing cardiovascular risk and contributing to clinical suspicion when associated with other characteristic features (43).

Etiologically, about 80% of cases are ACTH-dependent, with corticotroph pituitary adenomas being more common than ectopic ACTH-secreting tumors (43). ACTH-independent forms account for approximately 20% and include adrenal adenomas, carcinomas, and hyperplasias (43). Despite this heterogeneity, a common feature is the dysregulation of mechanisms controlling steroidogenesis. Among them, the cAMP–PKA pathway plays a central role in regulating cortisol secretion by adrenocortical cells (46, 47). Dysregulation of this signaling cascade has been documented in various forms of CS, contributing to aberrant cortisol secretion and emphasizing its relevance in the molecular pathogenesis of the disorder (**Figure 1B**).

#### 2.1.2 Genetic alterations in the cAMP-PKA axis

##### 2.1.2.1 GNAS

The *GNAS* gene, located on chromosome 20, encodes, among other products, the α-subunit of the stimulatory G protein (Gsα) (49). This protein is widely expressed and mediates the action of several hormones, neurotransmitters, and paracrine factors through the generation of the second messenger cAMP (49). Under physiological conditions, Gsα remains bound to GDP in a heterotrimeric complex with the β and γ subunits (49). Activation of G protein–coupled receptors induces the exchange of GDP for GTP and promotes the dissociation of Gsα from the Gβγ complex (49). Gsα then activates AC, which converts ATP into cAMP and triggers the release of the catalytic subunits of PKA, resulting in diverse intracellular responses (9, 13). The intrinsic GTPase activity of Gsα hydrolyzes GTP back to GDP, restoring the inactive state and ensuring that signaling remains transient and tightly regulated (49). Studies have shown that constitutive activation of Gsα enhances AC activity and aberrant cAMP synthesis, establishing a direct mechanism for autonomous steroidogenesis (43, 46).

MAS is the classic disorder associated with activating PV in *GNAS* (50). It results from postzygotic somatic mosaicism, in which mutated and normal cells coexist within different tissues, typically involving hotspot variants at Arg201 or Gln227 of *GNAS* gene (50). Clinically, MAS is characterized by the triad of café-au-lait skin pigmentation, polyostotic fibrous dysplasia, and precocious puberty, but it may also include cortisol excess, thyroid hyperfunction, and acromegaly, depending on the tissues affected (50, 51). In cases with adrenal involvement, autonomous cortisol secretion has been reported together with a distinctive histological pattern of bimorphic cortical hyperplasia, sustained by constitutive Gsα signaling in clusters of mutated cells (52). Because it arises from a PV acquired during postzygotic embryogenesis, MAS may present with CS already in newborns and children (29, 43). This condition is frequently associated with arterial hypertension and, in severe cases, may require bilateral adrenalectomy for hypercortisolism control (29, 51).

Beyond the syndromic context, activating *GNAS* PV have also been identified in acquired forms of ACTH-independent CS (33). In a cohort of five patients with CS due to primary bilateral macronodular adrenal hyperplasia (PBMAH), three carried somatic variants in this gene, including two p.R201H and one p.R201S variants in adrenal nodules, reinforcing the role of these alterations in adrenal tumorigenesis (33). None of these patients exhibited sufficient clinical features to support a diagnosis of MAS, representing the first reported cases of AIMAH caused by activating PV in the G protein. Another clinical case supporting the involvement of *GNAS* in the development of CS outside the syndromic setting described a patient with subclinical CS and bilateral functioning autonomous cortisol-secreting adrenocortical tumors, in whom a somatic p.R201H variant was identified in association with a paradoxical cortisol response to dexamethasone, indicating dysregulation of the cAMP-PKA pathway (53).

More recently, a novel somatic variant p.K58Q has been characterized in a cortisol-producing adrenocortical adenoma (32). Functional studies demonstrated that this alteration leads to increased basal cAMP levels, enhanced pCREB phosphorylation, and CRE-luciferase activation, in addition to influencing cell viability and apoptosis (32). These findings indicate a gain-of-function effect similar to Arg201 PV and broaden the mutational spectrum of *GNAS* in adrenal CS, underscoring that variants outside classical hotspots can also drive autonomous steroidogenesis through constitutive activation of the cAMP–PKA pathway (32).

##### 2.1.2.2 PRKAR1A

Among the regulatory subunits of PKA, RIα is distinguished by its almost ubiquitous distribution and by constituting the main component of type I PKA, the isoform most sensitive to cAMP and predominant in mediating this pathway in human cells (54, 55). The *PRKAR1A* gene, located on chromosome 17q22-24, encodes RIα and plays an essential role in maintaining the catalytic activity of the enzyme under cAMP control, ensuring that phosphorylation of target proteins occurs in a transient manner and adjusted to physiological demands (54). Alterations affecting this gene compromise this regulation, leading to constitutive activation of PKA and autonomous cortisol production, with direct repercussions on the development of endocrine hypertension (56–58).

CNC represents the main clinical condition associated with germline inactivating variants in *PRKAR1A*, showing autosomal dominant inheritance and high penetrance (14, 56). It is characterized by cutaneous pigmentation in lentigines, cardiac and cutaneous myxomas, and multiple endocrine and non-endocrine tumors (57, 59). Among the endocrine manifestations, PPNAD is the most common, present in about 25% of affected individuals, and can occur either in isolation or in association with CNC, being characterized by autonomous cortisol secretion and the development of ACTH-independent CS (56, 57, 59). Germline-inactivating variants of *PRKAR1A* are found in approximately 45 to 65% of patients with CNC, but this proportion can reach up to 80% in cases where PPNAD leads to CS, highlighting the central role of the gene in adrenal pathogenesis (60). Most of these alterations introduce premature termination codons, generally through nonsense or frameshift events, ultimately leading to loss of RIα expression (61). Furthermore, similar variants have also been described in sporadic or isolated forms of PPNAD, indicating that loss of RIα function constitutes a pathological mechanism both in familial disease and in non-syndromic cases (58, 60).

##### 2.1.2.3 PRKACA

In addition to the regulatory subunits, the catalytic ones also play a decisive role in the cAMP–PKA pathway, with the α isoform, encoded by the *PRKACA* gene, being the most relevant in the context of adrenal steroidogenesis (62). Located on chromosome 19p13.1, this gene spans approximately 26 kb organized into 10 exons and encodes a 351–amino acid protein, considered the main catalytic subunit of PKA and ubiquitously expressed in human tissues (37, 62). Under physiological conditions, Cα remains associated with the regulatory subunits, being released only after cAMP binding, which ensures that its kinase activity, responsible for phosphorylating target proteins on serine or threonine residues, occurs in a controlled and transient manner (37, 62). Alterations that disrupt this balance can lead to dissociation independent of cAMP and constitutive activation of Cα, promoting autonomous cortisol production and proliferative stimulus, thus constituting a central mechanism for the development of cortisol-producing adrenocortical tumors and endocrine hypertension (37, 38, 63, 64).

The main genetic alteration identified in *PRKACA* is the recurrent somatic PV p.L206R, described in approximately 20 to 70% of cortisol-producing adrenocortical adenomas, depending on the cohort (37, 62). This substitution is located in the p+1 loop region of the protein and compromises the interaction with the regulatory subunit RIα, preventing the stable formation of the holoenzyme and resulting in constitutive PKA activity (37, 62, 64). Functional studies in HEK293T cells demonstrated that this PV increases phosphorylation of downstream targets such as CREB and ATF1 by about 4 fold, thereby sustaining continuous steroidogenesis and autonomous cortisol secretion (37, 38).

Beyond the classical p.L206R, germline amplification of *PRKACA* has also been reported, leading to overexpression of the catalytic subunit and the development of PBMAH (65, 66). Additionally, studies in cortisol-producing adrenocortical adenomas demonstrated that PKA activation resulting from these PV directly correlates with overexpression of the steroidogenic acute regulatory protein (StAR), a key mediator of mitochondrial cholesterol transport and the rate-limiting step in steroidogenesis (31). A genotype–phenotype correlation has also been observed, with patients carrying *PRKACA* PV presenting with more severe hypercortisolism, earlier age at diagnosis, and generally smaller tumors compared with those without PV in this gene (65). These findings establish *PRKACA* as the most frequently altered gene in cortisol-producing adrenocortical adenomas, representing a central pathological mechanism in the development of ACTH-independent CS and its cardiovascular manifestations, including hypertension.

##### 2.1.2.4 *PDE11A* and *PDE8B*

The PDEs, already recognized as essential modulators of the cAMP–PKA pathway, also hold clinical relevance in the pathophysiology of adrenocortical tumors. Among the isoforms expressed in the adrenal cortex, *PDE11A* and *PDE8B* are noteworthy for their inactivating variants, which have been associated with micronodular hyperplasia exhibiting autonomous cortisol secretion (25, 26). Although less frequent than *PRKAR1A* or *PRKACA* PV, these alterations demonstrate that impaired degradation of cAMP results in constitutive activation of PKA, promoting steroidogenic autonomy and contributing to the spectrum of ACTH-independent CS, often accompanied by cardiovascular manifestations, including endocrine hypertension.

*PDE11A*, located on chromosome 2q31.2, encodes a dual-specificity enzyme capable of degrading both cAMP and cGMP, with the *PDE11A4* isoform being the predominant form expressed in adrenal tissue (26). In a 2006 study, germline variants in this gene were described in individuals with PPNAD and isolated micronodular adrenocortical hyperplasia associated with CS (26). These alterations resulted in loss of heterozygosity in the 2q31–2q35 region, accompanied by reduced *PDE11A4* expression and increased CREB phosphorylation, reflecting inefficient cAMP degradation and consequent sustained activation of the PKA pathway, thus establishing a direct mechanism of steroidogenic dysregulation. Subsequently, in an expanded cohort of patients with AIMAH, missense variants in *PDE11A* were identified in 28% of cases, compared with 7.2% of controls, confirming its association with genetic predisposition (21). Functional assays based on these findings demonstrated that substitutions such as D609N and M878V reduce enzymatic activity, leading to intracellular cAMP accumulation, enhanced CREB-dependent transcription, and an augmented forskolin-stimulated response in adrenocortical cells, consolidating the link between loss of enzymatic function and PKA hyperactivation.

More recently, a study involving patients with PBMAH demonstrated that deleterious *PDE11A* variants occur at similar frequencies among individuals with or without *ARMC5* PV, the main tumor suppressor gene implicated in this condition, being associated with a milder phenotype, characterized by lower cortisol levels and fewer adrenal nodules (67). These findings indicate that *PDE11A* functions as a phenotypic modifier and low-penetrance allele, rather than an isolated cause of disease.

*PDE8B*, in turn, located on chromosome 5q13.3, encodes a high-affinity, cAMP-specific phosphodiesterase that is abundantly expressed in the zona fasciculata of the adrenal cortex, where it regulates steroidogenesis (68). In 2008, Horvath and colleagues described a germline inactivating PV (c.914A>C, p.H305P) in a child with micronodular adrenocortical hyperplasia and CS, inherited from an asymptomatic parent with mild adrenal hyperplasia, suggesting an autosomal dominant inheritance with low penetrance (25). Functional assays in HEK293 cells transfected with this variant demonstrated intracellular cAMP accumulation and reduced enzymatic activity, indicating loss of function. Subsequently, missense and splicing variants in *PDE8B* were identified in PPNAD and cortisol-producing adrenocortical adenomas, all associated with increased cAMP levels and PKA hyperactivation (68, 69). In a transcriptomic analysis of unilateral adenomas, *PDE8B* showed the strongest positive correlation with cortisol secretion, reinforcing its role as a critical regulator of cAMP homeostasis and a modulator of PKA-dependent adrenocortical tumorigenesis (68).

### 2.2 Primary aldosteronism

#### 2.2.1 Clinical overview

PA represents the most frequent curable cause of secondary hypertension and is associated with a significant increase in cardiovascular morbidity and mortality (70). Initially considered a rare condition, it is now estimated to account for approximately 5–15% of hypertension cases, reaching 20–30% among patients with resistant hypertension (70, 71). The disease results from the autonomous production of aldosterone, independent of the renin–angiotensin–aldosterone system (RAAS), leading to sodium and water retention, expansion of extracellular volume, hypertension, and often hypokalemia (70). The consequent suppression of renin secretion, as a compensatory mechanism, results in an increased aldosterone-to-renin ratio, the principal biochemical marker of PA (70).

Under physiological conditions, aldosterone secretion by the zona glomerulosa is primarily regulated by potassium and angiotensin II but also undergoes acute modulation by ACTH (70, 72). ACTH acts on the melanocortin type 2 receptor (MC2R), stimulating the cAMP–PKA pathway and increasing the expression of *CYP11B2*, which encodes aldosterone synthase, in addition to enhancing steroidogenesis through the activation of regulatory proteins such as StAR (72, 73). In PA, aldosterone secretion occurs independently of the RAAS but can remain partially modulated by ACTH (72). Gene expression analyses of resected human aldosterone-producing adenomas and adjacent zona glomerulosa tissue have demonstrated *MC2R* overexpression, conferring an exaggerated response to ACTH and sustaining hormonal secretion via the cAMP–PKA pathway (72, 74, 75). This mechanism explains the morning elevation of aldosterone levels and their suppression following dexamethasone administration observed in patients with PA (75).

Beyond blood pressure elevation, aldosterone excess exerts deleterious effects on the heart, vessels, kidneys, and central nervous system, promoting left ventricular hypertrophy, myocardial fibrosis, endothelial dysfunction, renal hyperfiltration, and a higher incidence of stroke, atrial fibrillation, and metabolic alterations (70). These complications manifest more severely than would be expected from blood pressure elevation alone, highlighting the direct role of aldosterone in target-organ damage. Such alterations are largely reversible with specific treatment, either through unilateral adrenalectomy in cases of lateralized disease or through the use of mineralocorticoid receptor antagonists in bilateral forms (70, 71, 76). Despite its clinical relevance, PA remains underdiagnosed, with only about 1–2% of affected individuals being identified and treated during their lifetime, underscoring the importance of early diagnosis and targeted management (70, 71).

From a genetic perspective, recent advances have shown that most aldosterone-producing adenomas harbor somatic PV in genes regulating ion channels and calcium signaling, such as *KCNJ5*, *CACNA1D*, *ATP1A1*, *ATP2B3*, and *CTNNB1*, which promote membrane depolarization, calcium influx, and activation of steroidogenesis, ultimately leading to autonomous aldosterone secretion (70). In addition, rare familial forms have been described, caused by germline variants in *CYP11B1/CYP11B2* (FH-I), *CLCN2* (FH-II), *KCNJ5* (FH-III), and *CACNA1H* (FH-IV), generally inherited in an autosomal dominant pattern with variable penetrance (70, 76). These findings reinforce that PA represents a spectrum of genetic and acquired disorders whose molecular basis converges toward persistent activation of calcium- and cAMP-dependent pathways, promoting autonomous hormone secretion and adrenocortical remodeling.

#### 2.2.2 cAMP-PKA pathway alterations

In addition to their implication in the pathophysiology of CS, PDEs have also been investigated in studies focusing on PA. In this context, a recent work highlighted a possible correlation between *PDE2A* and *PDE3B* and the development of bilateral PA, raising the hypothesis that alterations in the signaling of the cAMP–PKA pathway, possibly modulated by ACTH-mediated stimuli, could contribute to autonomous aldosterone secretion (**Figure 1B**) (27). In patients with bilateral PA, Rassi-Cruz and colleagues identified rare germline variants in *PDE2A* (p.I629V) and *PDE3B* (p.R217Q and p.G392V), associated with autonomous aldosterone secretion and endocrine hypertension. Expression analyses revealed strong immunoreactivity of *PDE2A* in the normal zona glomerulosa and in hyperplastic areas positive for *CYP11B2*, whereas *PDE3B* showed moderate to strong diffuse expression in hyperplastic adrenal tissue. Functional evaluation demonstrated increased PKA activity in frozen tissues carrying the variants, consistent with cAMP accumulation due to inefficient degradation. Although *in vitro* assays using HEK293T cells showed reduced protein expression of the mutant enzymes without significant increase in PKA phosphorylation following cAMP stimulation, the tissue findings support a partial loss-of-function effect with functional impact in the adrenal tissue. Furthermore, in samples carrying *PDE3B* variants, increased expression of *SGK1* and *SCNN1G* (ENaCγ), genes regulated by aldosterone, was observed, indicating persistent activation of post-receptor pathways involved in sodium retention and increased blood pressure. These findings, together with the higher PKA activity detected in the affected tissues, suggest that *PDE3B* alterations contribute to cAMP–PKA pathway hyperactivation and autonomous aldosterone secretion. In contrast, the *PDE2A* variant did not show evident functional enhancement in cellular models, although the expression pattern observed in adrenal tissue supports a possible modulatory role of this enzyme in the zona glomerulosa.

### 2.3 Hypertension with brachydactyly

#### 2.3.1 Definition and clinical features

Hypertension with brachydactyly (HTNB), also known as Bilginturan syndrome, is a rare autosomal dominant condition characterized by the association of severe, salt-independent, and age-progressive hypertension with type E brachydactyly (24, 77, 78). Originally described in a Turkish family by Bilginturan and colleagues in 1973, the syndrome presents with symmetrical shortening of metacarpals, metatarsals, and phalanges, often accompanied by short stature and a rounded facial appearance (77, 78). The hypertensive phenotype manifests from childhood or adolescence and tends to worsen progressively, leading to early-onset cerebrovascular events that can be fatal if untreated (24, 78). Radiographic examinations reveal marked bone shortening, while laboratory and renal investigations are generally normal, reinforcing the non-renal and salt-independent nature of the hypertension (24, 78). Subsequent reports in different populations confirmed the autosomal dominant inheritance pattern and consistent clinical expression of the phenotype, characterizing HTNB as a distinct hereditary form of genetically based hypertension (24, 77).

#### 2.3.2 *PDE3A* and alterations in the cAMP–PKA pathway

Genetic and functional studies have consolidated the involvement of the cAMP–PKA pathway in the pathophysiology of HTNB (24, 79). Germline gain-of-function variants in *PDE3A*, first described by Maass and colleagues, increase the hydrolytic activity of the enzyme toward cAMP, thereby reducing the availability of this second messenger in vascular smooth muscle cells (**Figure 1C**) (24). This decrease in PKA-dependent signaling impairs cAMP-mediated vasodilatory mechanisms and promotes arterial remodeling, resulting in increased peripheral resistance and severe salt-independent hypertension. Subsequent studies demonstrated that dysfunction of the cAMP–PKA pathway in smooth muscle cells also affects cell growth regulation, contributing to medial thickening and the progressive hypertensive phenotype observed in variant carriers (79). Thus, although the cAMP–PKA pathway classically acts as a mediator of vascular relaxation, structural alterations in *PDE3A* convert its effect into a state of deficient signaling, establishing a model of hereditary hypertension resulting from reduced cAMP-dependent vasodilator tone.

## 3 Other endocrine causes of hypertension and their relationship with cAMP-PKA signaling

### 3.1 Acromegaly and gigantism

In acromegaly and gigantism, chronic excess of growth hormone (GH) and insulin-like growth factor 1 (IGF-1) contributes to arterial hypertension through a multifactorial process involving sodium and water retention, plasma volume expansion, endothelial dysfunction, sympathetic activation, and vascular remodeling (80). At the molecular level, constitutive activation of the cAMP–PKA pathway represents a key driver of somatotroph tumorigenesis in selected genetic contexts, including *GNAS*-related disorders such as MAS, *PRKAR1A*-associated CNC, and conditions involving enhanced cAMP signaling through aberrant GPCR activation (50, 81, 82). In these settings, sustained GH hypersecretion primarily results from the direct effects of these molecular alterations on pituitary cell proliferation and function.

### 3.2 Hyperthyroidism

In hyperthyroidism, elevated circulating thyroid hormone levels exert profound cardiovascular effects, including increased heart rate, enhanced myocardial contractility, expanded blood volume, and reduced systemic vascular resistance, resulting in a hyperdynamic circulatory state and predominantly systolic hypertension (83). These hemodynamic alterations are mediated by both genomic and non-genomic actions of thyroid hormones, as well as by heightened sensitivity to catecholamines and functional activation of adrenergic signaling pathways. At the cellular level, thyroid-stimulating hormone receptor (TSHR) signaling is physiologically coupled to Gs-mediated activation of adenylate cyclase in thyroid follicular cells, while experimental evidence obtained in murine models further indicates that β-adrenergic receptor–dependent pathways engage cAMP–PKA signaling to promote cardiac remodeling and functional adaptation under conditions of thyroid hormone excess (84). In addition, somatic activating variants in *TSHR* and *GNAS* have been described in 85% of hyperfunctioning thyroid nodules, leading to constitutive Gs-mediated activation of the cAMP-PKA pathway and contributing to autonomous thyroid hormone production (85).

### 3.3 Pheochromocytoma and paraganglioma

In pheochromocytoma and paraganglioma, arterial hypertension arises predominantly from excessive catecholamine secretion, which induces sustained vasoconstriction, increased cardiac output, and heightened sympathetic tone (86). At the molecular level, tumorigenesis is mainly driven by alterations in hypoxia signaling, mitochondrial metabolism, and kinase pathways, including pathogenic variants in genes such as *SDHx*, *VHL*, *EPAS1*, *RET*, and *NF1*, among other driver genes (86, 87). In pheochromocytomas and paragangliomas, hypertension arises predominantly as a downstream consequence of activation of α1-adrenergic receptors by epinephrine and norepinephrine, rather than from direct molecular disturbances within the cAMP–PKA signaling cascade, reflecting a fundamentally different pathophysiological framework. Accordingly, in this context, hypertension reflects the systemic cardiovascular consequences of catecholamine excess rather than intrisic defects of intracellular cAMP–PKA signaling.

## 4 Final remarks

The evidence summarized in this review underscores the pivotal role of the cAMP–PKA signaling pathway as a unifying molecular framework linking endocrine and non-endocrine forms of hypertension. Germline and somatic pathogenic variants affecting genes that regulate cAMP synthesis, degradation, or PKA activation—such as *GNAS*, *PRKAR1A*, *PRKACA*, *PDE11A*, *PDE8B*, *PDE2A*, and *PDE3B*—disrupt the finely tuned balance of intracellular signaling that governs adrenal steroidogenesis and vascular tone. These genetic alterations collectively illustrate how both excessive and deficient cAMP–PKA signaling can converge on hypertensive phenotypes through distinct pathophysiological mechanisms.

In the adrenal cortex, constitutive activation of the cAMP–PKA cascade drives autonomous cortisol or aldosterone secretion, contributing to disorders such as CS and primary aldosteronism. Conversely, in vascular smooth muscle, gain-of-function variants in *PDE3A* lead to excessive cAMP hydrolysis, impairing vasodilatory responses and resulting in the severe, salt-independent hypertension observed in hypertension-brachydactyly syndrome. These apparently opposite mechanisms—hyperactivation versus inactivation—highlight the dual and context-dependent role of cAMP signaling in blood pressure regulation.

In this context-dependent framework, the therapeutic implications of targeting PDEs deserve careful consideration. In most endocrine forms of hypertension driven by constitutive hyperactivation of the cAMP–PKA pathway, pharmacological inhibition of PDE activity is mechanistically counterintuitive, as it would be expected to further amplify intracellular cAMP signaling, potentially exacerbating autonomous steroidogenesis and hormonal excess. This paradox likely explains why PDE inhibitors have not found a role in the routine management of endocrine hypertension, in contrast to mineralocorticoid receptor antagonists and other agents targeting downstream hormonal effects (88). Notably, however, an opposite rationale applies to hypertension with brachydactyly syndrome, in which gain-of-function variants in *PDE3A* lead to excessive cAMP hydrolysis and impaired vasodilatory signaling. In this specific vascular context, selective PDE3 inhibition may theoretically restore intracellular cAMP levels and improve vascular relaxation, representing a potential precision-based therapeutic strategy that warrants further experimental and clinical investigation (89). These opposing mechanisms are summarized in **Figures 1B, C**, respectively.

The progressive elucidation of these mechanisms has reshaped the understanding of endocrine hypertension, revealing that subtle disturbances in intracellular messenger homeostasis may underlie conditions previously considered sporadic or idiopathic. Importantly, the identification of pathogenic variants within this pathway provides new perspectives for precision medicine, enabling molecular diagnosis, risk stratification, and the development of targeted therapies. Pharmacologic modulation of PKA activity, selective phosphodiesterase inhibition, and correction of receptor-mediated signaling abnormalities represent promising therapeutic avenues currently under exploration.

Continued integration of genomic, transcriptomic, and functional analyses will be essential to delineate genotype–phenotype correlations and uncover additional regulatory components of the cAMP–PKA network. Such advances will deepen our understanding of the molecular determinants of hypertension and steroidogenic autonomy, ultimately translating into more effective and individualized clinical interventions.
